# Breakthrough Acute HIV Infections among Pre-Exposure Prophylaxis Users with High Adherence: A Narrative Review

**DOI:** 10.3390/v16060951

**Published:** 2024-06-12

**Authors:** Davide Moschese, Samuel Lazzarin, Martina Laura Colombo, Francesco Caruso, Andrea Giacomelli, Spinello Antinori, Andrea Gori

**Affiliations:** 1 I Division of Infectious Diseases, ASST Fatebenefratelli Sacco, Luigi Sacco Hospital, 20157 Milan, Italy; andrea.gori@unimi.it; 2 Department of Biomedical and Clinical Sciences, Università degli Studi di Milano, 20133 Milan, Italy; samuel.lazzarin@unimi.it (S.L.); martinalaura.colombo@unimi.it (M.L.C.); francesco.caruso@unimi.it (F.C.); dott.giacomelli@gmail.com (A.G.); spinello.antinori@unimi.it (S.A.); 3 III Infectious Diseases Unit, ASST Fatebenefratelli Sacco, Luigi Sacco Hospital, 20157 Milan, Italy; 4 II Infectious Diseases Unit, ASST Fatebenefratelli Sacco, Luigi Sacco Hospital, 20157 Milan, Italy; 5 Centre for Multidisciplinary Research in Health Science (MACH), Università degli Studi di Milano, 20122 Milan, Italy

**Keywords:** HIV, primary HIV, PrEP, failure, adherence

## Abstract

Pre-exposure prophylaxis (PrEP) is a pivotal intervention among HIV prevention strategies. We aimed to narratively revise the topic of HIV acute infection in the setting of PrEP exposure with a focus on diagnostic options, clinical features, and future PrEP perspectives, with a particular focus on users with high adherence to PrEP. We searched the main databases (PubMed, Embase, and Scopus) with the keywords “PrEP” or “Pre-Exposure Prophylaxis” and “HIV” or “PLWH” and “breakthrough” or “acute infection” or “primary infection”. We included all randomized clinical trials and non-experimental studies (both case reports and observational studies) ever published. In the present narrative review, we revise the diagnostic challenges related to HIV diagnosis in the setting of PrEP and the clinical characteristics and symptoms of breakthrough infections. We discuss the management of acute HIV infection during PrEP and the new challenges that arise from the use of long-acting drugs for PrEP. Our review underlines that although extremely rare, HIV seroconversions are still possible during PrEP, even in a context of high adherence. Efforts to promptly identify these events must be included in the PrEP follow-up in order to minimize the chance of overlooked HIV breakthrough infections and thus exposure to suboptimal concentrations of antiretrovirals.

## 1. Introduction

Forty years since the first reports of AIDS, the therapeutic advancements in the field of HIV have granted people with HIV (PWH) a life expectancy similar to that of people without HIV and an improved quality of life [1]. The key factor to successful treatment is an early diagnosis with a prompt start of antiretroviral treatment. This is pivotal for both overall mortality and comorbidity reduction and to reduce the risk of transmission. In particular, an international cornerstone study, the INSIGHT START study, showed how patients who started antiretroviral treatment had a significantly lower risk of developing severe AIDS-related events compared to patients who delayed the start of treatment, regardless of their CD4 cell count [2,3]. Despite these advancements, however, new HIV acquisitions are still a public health challenge worldwide. In particular, despite the different preventive strategies that are available nowadays, recent data from UNAIDS show that we are still far from the end of the AIDS pandemic [4].

The use of pre-exposure prophylaxis (PrEP) has quickly arisen as a major player in the fight against the ongoing HIV pandemic. The first pieces of evidence of the effectiveness of oral chemoprophylaxis in reducing the risk of HIV transmission date back to over a decade ago, as trials showed high-level protection from the infection while regularly consuming tenofovir disoproxil fumarate (TDF) and emtricitabine (FTC) when compared to a placebo [5]. As proven by a recent meta-analysis, PrEP has been shown to be particularly effective in men who have sex with men (MSM), with a rate reduction of 75% of new infections; furthermore, the meta-analysis highlighted how adherence was a keystone for treatment success, with the rate of reduction rising up to 86% in trials with high adherence [6].

From the first commercial release of oral PrEP in 2012, its use has steadily increased over the years, becoming widely accessible in most high-income and some lower-income countries [4]. While oral tablets are, as of now, the main available tool for chemoprophylaxis against HIV, new options are gaining popularity, such as dapivirine monthly rings, studied for the female population, and cabotegravir-based long-acting PrEP [7,8].

Although PrEP is highly effective, seroconversions have been observed with a variable incidence in trials [9,10]. Failure could be a consequence of an unrecognized infection acquired before the initiation of PrEP, low adherence to the oral regimen, or the acquisition of a virus with resistance mutations to PrEP drugs [11,12].

A universal consensus on the definition of “PrEP failure” is still lacking, though it has been described by some authors as “all seroconversions occurring at any time along the care continuum for PrEP” [13]. The absence of a clear and consistent definition does not thus fully allow for the comparison of different reports.

We aimed to narratively revise the topic of HIV acute infection (AHI) in the setting of PrEP exposure with a focus on diagnostic options, clinical features, and future PrEP perspectives with a particular focus on users with high adherence to PrEP.

## 2. Materials and Methods

We searched the main databases (PubMed, Embase, and Scopus) with the keywords “PrEP” or “pre exposure prophylaxis” and “HIV” or “PLWH” and “breakthrough” or “acute infection” or “primary infection” with the aim of producing a narrative review of PrEP breakthrough infections. We included all randomized clinical trials and non-experimental studies (both case reports and observational studies) ever published. Three reviewers (SL, MLC, and FC) independently screened the titles and abstracts to determine eligibility for full-text review. No geographical restrictions were applied. Only publications in peer-reviewed journals in the English language were included. Studies were included if they met all of the following criteria: (i) study was published in full; (ii) study described PrEP; and (iii) study included any kind of PrEP, such as TDF, TDF/FTC, TAF/FTC, dapivirine ring, and long-acting cabotegravir. Conference papers and abstracts were excluded. Among those, we only selected papers featuring the characteristics of PrEP users with a breakthrough of acute HIV infection with a focus on adherence, diagnostic tools, and symptoms reported.

## 3. Results

### 3.1. Diagnostic Tools

HIV early diagnosis has always proven to be a challenge, both from a clinical and a technical point of view. As a variety of new diagnostic tools has been developed over time, each with its own target and sensitivity, it has become crucial for the physician to know which test to use considering the possible timing of infection.

Highly sensitive immunoassays targeting p24 antigen and anti-HIV antibodies represent the standard of care for HIV screening in high-income countries, whereas Western blot (WB) is currently used as the main confirmatory assay [14,15]. Aside from serological tests, nucleic acid amplification tests (NAATs) represent, as of now, the gold standard in HIV viral load testing, which is a key prognostic marker for disease progression and the indicator of response to antiretroviral therapy (ART).

In regard to early infection, Fiebig staging provides a descriptive tool based on antigen p24, WB, and HIV-RNA, where each stage describes a unique pattern of assay reactivity [16]. Stage 0, or the eclipse period, is characterized by completely undetectable viral markers in blood samples, as it corresponds to the earliest phase of infection, lasting on average 5 to 7 days [17,18]. During stage I, HIV-RNA becomes detectable, as all other tests remain negative; during stage II, p24 antigen is also detectable, in addition to NAATs; stage III is characterized by positivity of HIV-RNA, p24 antigen, and IgM-sensitive assays, even though WB is still negative; in stage IV, WB shows an indeterminate pattern, in which the first HIV-specific bands are detectable, though failing to meet the international interpretative criteria of positivity (i.e., at least two of p24, gp41, or gp120/160 are reactive); stage V is characterized by a positive pattern, lacking p31 reactivity; lastly, in stage VI, WB displays a fully reactive pattern, which includes a p31-specific band (this stage indicates an infection acquired within the previous 2 to 3 months) [16,19,20].

Understanding the dynamic of serological and virological assays in the context of PrEP exposure is crucial for an adequate management of people with a suspected AHI while taking PrEP. Indeed, the results of the above-mentioned tests have shown to be influenced by external factors. The PARTNERS study, a retrospective analysis of a controlled, double-blind, randomized trial of PrEP (TDF/FTC or TDF alone) compared to a placebo, observed a significant increase in the mean time to a positive Western blot in the PrEP group, estimated as 80 vs. 49 days. The same study showed that HIV-RNA viral load was on average 2 or 3 log10 lower in those with AHI in the PrEP group when compared to a placebo [21]. These findings suggest that PrEP might delay seroconversion and even hinder HIV-RNA detection during the acute phase of infection, making the diagnosis of AHI a challenge [14].

It is crucial to rule out AHI before PrEP start, as exposure to TDF/FTC in unrecognized HIV infection could make diagnosis more difficult while failing to adequately control viremia. It has also been reported that suboptimal exposure to antiretrovirals carries the potential to select resistance mutations [22]. In light of this, most international guidelines emphasize the HIV testing algorithm to be implemented in the context of PrEP initiation. Furthermore, people taking PrEP are supposed to be monitored for HIV with trimestral testing [23].

### 3.2. AHI Clinical Spectrum

AHI can be clinically apparent as a mononucleosis-like illness, which can be referred to as “acute retroviral syndrome”. It consists of fever, pharyngitis, generalized lymphadenopathy, weight loss, gastrointestinal manifestations, and a maculopapular, urticarial, or roseola-like rash. Less commonly, a neurological syndrome, such as aseptic meningitis, encephalitis, or peripheral neuropathy with Guillain–Barré syndrome, can be observed. Conversely, opportunistic infections have been seldom described, such as *Candida* spp. esophagitis or *P. jirovecii* pneumonia [24].

Symptoms of acute retroviral syndrome usually develop 2 to 4 weeks after HIV exposure (longer timeframes, reaching 10 weeks, have also been described), generally last for 10 to 15 days, and mostly resolve spontaneously [19,24]. The clinical manifestation of infection usually precedes peak viremia, typically in a stage in which anti-HIV antibodies have not yet been developed and even p24 is still undetectable in blood. HIV-RNA NAAT is the assay of choice, which allows for detection of the typical high-level viremia observed in this phase. The actual prevalence of symptoms in acutely infected people is still a matter of debate, as most asymptomatic infections remain undetected. The estimated proportion of symptomatic AHI varies widely, typically ranging from 10 to 60% [24,25].

**Figure 1:**
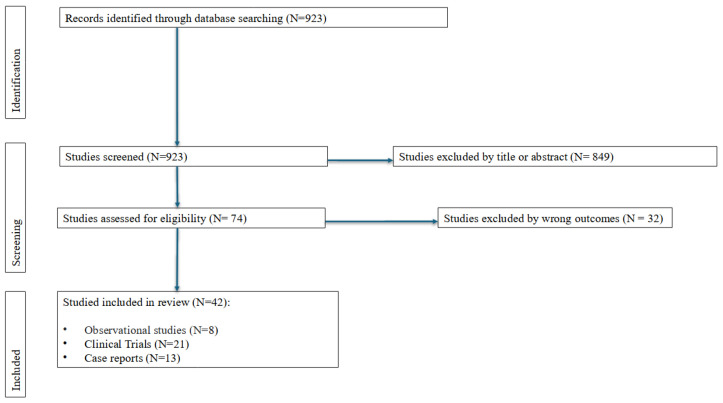
Study identification and selection process.

A total of 42 studies were included in the final analysis of this narrative review. Among them, 13 were case reports, 8 were observational studies, and 21 were clinical trials. The full selection process is represented graphically in Figure 1.

### 3.3. Breakthrough Acute HIV Infections Reported in Experimental and Non-Experimental Studies

We identified 13 published case reports describing 15 cases of AHI that occurred during PrEP care (Table 1).

Among these cases, 13 individuals were on a TDF/FTC regimen (12 daily, 1 on-demand), while the remaining 2 were on a TDF-alone regimen for HBV-related chronic hepatitis. Most AHIs were identified using a fourth-generation assay with or without HIV-RNA NAAT, with five patients showing clinical signs or symptoms of AHI. Adherence was evaluated using various methods, including therapeutic drug monitoring (TDM), dried blood spot (DBS), hair analysis, pill dispensation records, or self-reporting. By applying a definition of true PrEP failures including any AHI in patients with documented consistent adherence to PrEP, we identified seven cases of true PrEP failure. Of note, three additional cases were consistent with HIV infection acquired prior to the initiation of PrEP in a context of good PrEP adherence.

Regarding the data from observational studies, we identified eight studies that collectively reported 315 cases of seroconversions among a total of 45,947 participants (Table 2).

Remarkably, a single study contributed 266 of them, yet it did not assess adherence, leaving 49 seroconversions with available adherence data. Among these, 23 were identified as true PrEP failures. Data regarding clinical manifestations were only available in two of the reviewed studies.

Finally, 21 clinical trials were analyzed, documenting 389 seroconversions among a total of 38,945 participants (Table 3).

### 3.4. Seroconversions versus True Breakthrough Infections in Clinical Trials

All clinical trials included an adherence assessment. After excluding cases where patients were considered non-adherent to PrEP, a total of 55 AHI were categorized as true PrEP failures. Data regarding clinical manifestations were only available in one of the reviewed studies.

Among all of the selected studies, the proportion of seroconversions in oral TDF/XTC PrEP users was 260/30,563 (0.85%), with breakthrough infections in oral TDF/XTC PrEP users (total of cases with adherence available data) accounting for 0.08% (22/28,366).

**Figure 2:**
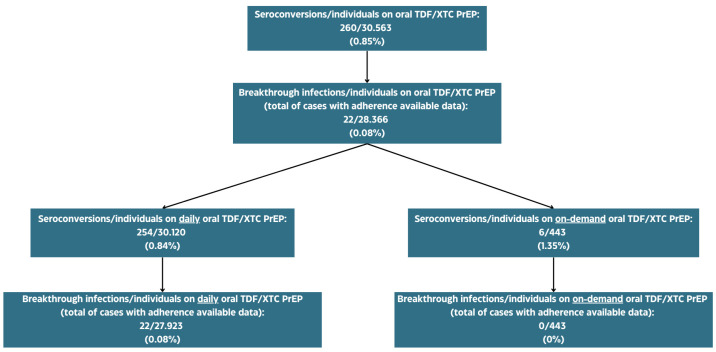
Rate of seroconversions and true breakthrough infections among oral PrEP users in clinical trials.

Focusing on those consuming oral TDF/XTC PrEP with a daily scheme, the overall seroconversions were 254/30,120 (0.84%), with a similar breakthrough infection rate of 22/27,923 (0.08%), while those consuming oral TDF/XTC PrEP with an on-demand scheme had a higher rate of seroconversion (6/443; 1.35%), but no breakthrough infections were reported in this group (Figure 2).

Analyzing topical PrEP (either with tenofovir 1% gel and dapivirine vaginal ring) in the sole context of clinical trials, a total of 42/638 (6.58%) of seroconversions were observed, with 9/638 (1.41%) breakthrough infections.

Only one trial featuring injectable CAB PrEP reported both seroconversions (16/4566; 0.35%) and breakthrough infections (4/4566; 0.09%).

We need to be clear that besides clinical trials, the majority of the studies had a measurement of adherence based on patients’ self-reports (Table 1 and Table 2).

### 3.5. How to Minimize the Risk of Overlooked HIV Infection during PrEP Start

A crucial step for clinicians before prescribing PrEP is the assessment of HIV status, as, in that moment, the exclusion of unrecognized AHI is of pivotal importance. Recent studies have shown that starting PrEP during an undiagnosed AHI is the main driver of selection for HIV drug resistance, which could potentially complicate subsequent HIV management [66,67,68,69,70,71].

**Figure 3:**
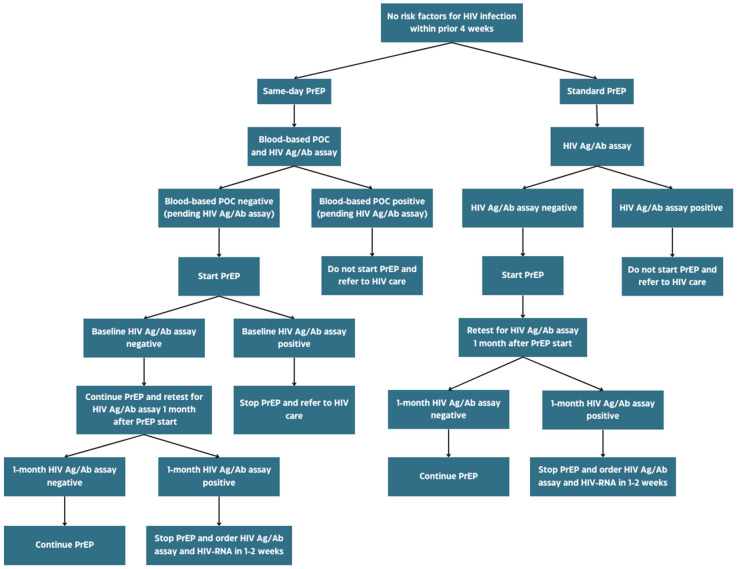
Decision algorithm for PrEP start in the absence of risk factors for recent HIV infection. POC: point-of-care.

Most international guidelines recommend as a best choice for HIV testing a fourth-generation antigen/antibody assay conducted by a laboratory and obtained within one to four weeks before initiating PrEP. Repeating the test after one month should also be considered to exclude inadvertent AHI at PrEP start. According to US Public Health Service and BHIVA/BASHH guidelines, a negative blood-based point-of-care (POC) test is acceptable for same-day PrEP initiation, but a laboratory fourth-generation antigen/antibody assay should always be ordered at baseline so that in case of unrecognized AHI the patient can be rapidly transitioned from PrEP to HIV care [72,73,74,75] (Figure 3).

This timely transition is essential in light of recent evidence suggesting that the M184 mutation can develop more rapidly than thought before, within just 1–2 weeks of TDF/FTC exposure [66].

**Figure 4:**
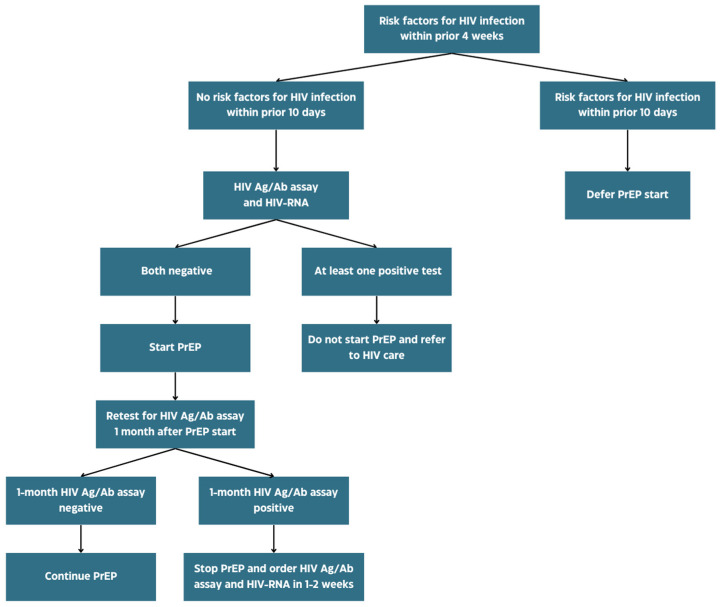
Decision algorithm for PrEP start when at least a risk factor for recent HIV infection is present. Of note, a positive HIV-RNA NAAT with a number of copies per milliliter close to the limit of detection should be interpreted cautiously, as false positive cannot be excluded; in such cases, a confirmatory HIV-RNA on a new plasma sample should be ordered.

The risk factors for AHI should be investigated during every initial evaluation for PrEP. Everyone should thus be questioned about engagement in risky behaviors and the presence of signs or symptoms consistent with AHI in the prior 4 weeks. If any of these risk factors are reported, a plasma sample for HIV-RNA NAAT should be sent to increase sensitivity in AHI diagnosis [72,73,74,75]. However, it is largely known that during the first 7 to 10 days after HIV infection (the eclipse phase), even HIV-RNA could be undetectable [76]. It seems reasonable, then, to defer PrEP start and retest after 2–4 weeks with both a fourth-generation antigen/antibody assay and HIV-RNA NAAT in case of very recent at-risk exposure or onset of AHI clinical features. Although highly sensitive, HIV-RNA testing is more expensive and not fully free from false positive results, which are likely to be the consequence of a laboratory error [77,78]. In such cases, low viral loads are usually detected, and repeating HIV-RNA NAAT on a new plasma sample is recommended by US Public Health Service guidelines in the case of viral loads below 200 copies per milliliter [72]. Since the occurrence of false positives cannot be excluded, we also suggest that a low-level viremia on a single plasma sample, without a complementary positive fourth-generation test, should prompt confirmation with an HIV-RNA NAAT on a further plasma sample (Figure 4).

Although there is a potential greater risk for NRTIs’ Resistance-Associated Mutations (RAMs) insurgence with the use of self-prescribed PrEP in unrecognized HIV infections, a recent experience highlighted how the consistent implementation of self-testing in this population overcame the risk for RAMs selection in community-based services. Given this, none of the included studies reported a breakthrough HIV infection during self-prescribed PrEP [79].

### 3.6. How to Manage Breakthrough AHI

Quarterly HIV testing is required for all PrEP users, which allows for timely diagnosis and treatment of breakthrough HIV infections. However, there is increasing evidence that exposure to antiretrovirals used as PrEP at the time of infection may alter the dynamics of viremia and the patient’s immune response [80]. Indeed, AHI in PrEP users usually presents with a lower viral load peak and set point and a prolonged seroconversion period, which might be delayed by several weeks. Antibody development may occur out of synchrony, with detectable antibodies in the absence of detectable antigens. Consequently, HIV testing during follow-up visits may yield ambiguous results, which, although infrequent, may be responsible for either delay in accurate diagnosis (falsely negative) or psychological stress (falsely positive) [81].

Because ambiguous results are often due to very early infection or technical issues, a reasonable strategy to confirm the presence or absence of infection is repeat testing in a few days or weeks [81].

Some authors recommend adherence-driven management of patients while their HIV status is being confirmed; if adherence is high, continuing PrEP may be the best decision, whereas in the case of inconsistent adherence, a timely transition to ART may be reasonable [81]. These strategies are based on the supposed pre-test probability of AHI according to different levels of adherence to PrEP, but they are both characterized by ongoing antiretroviral use, which could suppress viral replication, thus making HIV diagnosis more difficult [81]. Moreover, continuous antiretroviral exposure and limited viral replication would make it arduous to perform a genotypic resistance testing, which is essential in the subsequent management of breakthrough AHI. This appears even more true if we consider the significant rate of transmitted drug resistance observed in previous studies.

Testing for HIV-DNA might be helpful, but its use is currently limited to the research field at selected laboratories. Furthermore, it is known that viral seeding of peripheral blood mononuclear cells (PBMCs) occurs in the earlier stages of infection, and this phenomenon could be hindered by early exposure to antiretrovirals [82]. Consequently, HIV-DNA assays may yield false negative results in AHI in people taking PrEP.

In this context, discontinuation of PrEP for 1–2 weeks may be resolutive, allowing for viral replication in infected patients. This approach, though operationally simple, has two main pitfalls. First, if the patient is uninfected, there is a higher risk of infection in the case of ongoing sexual risk exposure; second, in the case of unrecognized infection, there is a reduction in viro immunological benefits related to rapid diagnosis and treatment, as well as in prevention benefits against onward transmission [81]. However, we believe that the transient use of other HIV-prevention strategies (condom, sexual abstinence), along with appropriate counselling, would reduce both the risk of infection in uninfected patients and the risk of onward transmission in patients with unrecognized AHI. PrEP discontinuation in infected patients would also allow for a sufficient viral replication to perform genotypic resistance testing, which would guide future treatment choices.

According to BHIVA/BASHH guidelines, TDM for tenofovir and emtricitabine should be considered to assess adherence [73]. However, TDM only offers insight into recent dosing, and it does not provide reliable information about cumulative dosing adherence, which can be evaluated through more advanced but costly and poorly available methods of drug analysis (tenofovir diphosphate level in red blood cells on DBS, hair analysis and segmental hair analysis) [11]. Nonetheless, despite providing a better understanding of the mechanisms underlying AHI during PrEP, pharmacology alone is currently insufficient to inform treatment decisions.

Once HIV infection is established, there are three key factors to be considered, namely timing of acquisition, level of adherence, and risk of resistance mutations. If HIV infection occurs before PrEP start, the risk of drug resistance is the highest, while it is generally lower when HIV infection is acquired after PrEP start. In the setting of optimal adherence to PrEP, breakthrough infections are extremely rare and usually associated with PrEP-resistant HIV strains [66,67,68,69,70,71]. Conversely, in the setting of poor adherence to PrEP, HIV infection is more frequent, but drug resistance is uncommon because of insufficient drug pressure [80].

Regardless of the specific pathway of infection, which is difficult to identify in daily clinical practice, HIV treatment should be initiated while waiting for the results of baseline resistance testing. Most guidelines recommend upgrading the TDF/FTC PrEP regimen to a three-drug regimen by including a third drug with a high genetic barrier to resistance (dolutegravir, bictegravir, or boosted darunavir) [72,73,74,75]. Despite limited evidence, such regimens are preferred as they seem to be effective even in the case of drug resistance mutations (M184I/V and K65R, conferring resistance to FTC and TDF, respectively). Two-drug regimens, such as dolutegravir plus lamivudine, are currently not recommended for first-line therapy, as data on breakthrough infections are lacking [83].

## 4. Future Perspectives in the Era of Long-Acting PrEP

Recently, new PrEP options with novel administration modalities have been proposed in an attempt to overcome adherence issues in people struggling with daily pill regimens [84]. Cabotegravir long-acting (CAB-LA), given as intramuscular injections every 2 months, proved to be superior to oral PrEP in different target populations and was approved by the US Food and Drug Administration in December 2021 [62,85].

However, some concerns have been raised about HIV infections acquired during CAB-LA PrEP or before its start but unrecognized. Although rare, new HIV infections in this setting have a different clinical and virological presentation from AHI, which is often symptomatic and readily detectable with traditional laboratory assays. Long-acting early viral inhibition (LEVI) syndrome is the term coined to describe the unique characteristics of new HIV infections in the setting of CAB-LA PrEP, including smoldering viral replication, delayed detection with traditional fourth-generation assays, increased risk of drug resistance, and minimal or no symptoms [62]. The observed increased risk of resistance to integrase-strand-transfer inhibitors (INSTI) is particularly troubling, as most international guidelines recommend INSTI-based regimens as the first-line treatment for new HIV infections [8,74,75,86].

In this uncharted scenario, HIV screening strategies should be adjusted, and consistent evidence supports the US Public Health Service guidelines, which recommend the use of HIV-RNA testing within one week before starting CAB-LA PrEP, at every injection visit, and quarterly for 12 months after stopping injections [72]. Sensitive RNA assays, indeed, detect most new infections before major INSTI resistance mutations develop [87]. However, the cost-effectiveness of HIV-RNA testing is yet to be determined, and the limited availability and higher costs of this screening strategy would probably prevent wide access to CAB-LA PrEP, especially in low- and middle-income countries. It is noteworthy that in the setting of CAB-LA PrEP implementation without NAAT, the predicted higher risk of INSTI resistance should be balanced with the significant decline in new HIV infections [88,89].

Prolonged screening after injection discontinuation is required because of the risk of drug-resistant HIV infection during the so-called “tail period”, during which cabotegravir plasma concentrations drop under protective levels, though maintaining potential for selective pressure. For the same reason, people with ongoing risk of HIV exposure should be offered daily oral PrEP during this time [84].

As for the treatment of new HIV infections, US Public Health Service guidelines suggest avoiding INSTI-based regimens and recommend the initiation of a three-drug regimen with boosted-darunavir, pending the results of baseline genotypic resistance testing [8].

Further studies are still needed to evaluate the feasibility of HIV-RNA screening on a large scale and to identify optimal treatment regimens for breakthrough infections.

## 5. Conclusions

In conclusion, although rare, HIV breakthrough acute infections could also occur in the setting of a high adherence to PrEP. Thus, physicians should be aware of the challenges related to the diagnosis and about the possibility of drug resistance mutations’ selection in case of suboptimal antiretroviral pressure, such as that of PrEP exposure with an ongoing HIV infection. In a future scenario with a potential large scale-up of long-acting PrEP, new challenges would probably arise, and questions regarding the optimal management of long-acting PrEP users are still open.

## Figures and Tables

**Table 1:** Case reports reporting breakthrough HIV infection in PrEP users.

Study	Year of Pub.	Location		Characteristics	PrEP Regimen	Time Using PrEP	Seroconversion	Resistance	True PrEP Failure
Ivan Chivite et al. [26]	2022	Spain	Patient 1	Age 23 y Male Sex worker Chemsex user	Oral TDF/FTC Daily	6 months	Time between test negative and positive: 180 days 4th-generation test and HIV-RNA positive	M184V K103N	No: irregular adherence to PrEP
			Patient 2	Age 35 y Male	Oral TDF/FTC Daily	30 days	Time between test negative and positive: 45 days 4th-generation test and HIV-RNA positive	M184V M184I	No: likely already infected at time of PrEP start
Volk J.E.A. et al. [27]	2018	San Francisco	Patient 1	Age 23 y MSM History of IDU	Oral TDF/FTC On demand	1 year	Time between test negative and positive: 1 year 4th-generation test and HIV-RNA positive	M184V	No: poor adherence to PrEP
Hoornenborg E. et al. [28]	2017	Amsterdam	Patient 1	Age 50 y MSM	Oral TDF/FTC Daily	8 months	Time between test negative and positive: unknown WB with only gp160 detected PCR for HIV-RNA and DNA on bulk PBMCs and sigmoid biopsies were negative HIV-RNA undetectable at diagnosis, but detectable after 3 weeks of PrEP withdrawal	Wild-type	Yes: good adherence; TDF concentrations in blood were stable and high
Naicker C.L. et al. [29]	2020	South Africa	Patient 1	Age 20 y Woman in a serodiscordant relationship	Oral TDF/FTC Daily	9 months	Time between test negative and positive: 9 months AHI symptoms 3rd-generation test and HIV-RNA positive Retrospective HIV viral load testing was conducted on all available stored samples and showed an increasing HIV viral load trend from month one of PrEP exposure	M184V K65R	No: likely already infected at time of PrEP start
Fox J. et al. [30]	2016	London	Patient 1	MSM Chronic HBV infection	Oral TDF monotherapy (HBV) Daily	4 years	Time between test negative and positive: 12 days AHI symptoms 4th-generation test and WB with 3 reactive bands; HIV-RNA undetectable	Wild-type	NA
			Patient 2	MSM Chronic HBV infection	Oral TDF monotherapy (HBV) Daily	3 years	Time between test negative and positive: unknown AHI symptoms 4th-generation test and HIV-RNA positive	Wild-type	NA
Colby D.J. et al. [31]	2018	Thailand	Patient 1	Age 28 y Sex worker	Oral TDF/FTC Daily	8 weeks	Time between test negative and positive: NA 3rd-generation test negative and HIV-RNA positive	M184V A98G K103N	No: likely already infected before PrEP start
Lee S.-S. et al. [32]	2020	Hong Kong	Patient 1	Age 24 y MSM Chemsex	Oral TDF/FTC	6 weeks	Time between test negative and positive: 6 weeks 4th-generation test, WB, and HIV-RNA positive	M184 V	NO: suboptimal adherence
Knox D.C. et al. [12]	2017	Toronto (Canada)	Patient 1	Age 43 y MSM	Oral TDF/FTC Daily	24 months	Time between test negative and positive: 3 months 4th-generation test positive and WB negative	INSTI: 51Y, 92Q RT: 41L, 67G, 69D, 70R, 184V, 215E; 181C	Yes: good adherence; TDF level in hair was consistent with long-term adherence and pharmacy dispensation records
Thaden J.T. et al. [33]	2018	North Carolina	Patient 1	Age 34 y MSM	Oral TDF/FTC Daily	14 months	Time between test negative and positive: unknown AHI symptoms 1 month before the test (fevers, chills, myalgias) 4th-generation test positive and HIV-RNA positive	M184V, K70T, K65R, and K103N	Yes: good adherence; plasma TDF and FTC concentrations were consistent with recent dosing and hair drug levels were commensurate with consistently high PrEP adherence over the prior 3 months
Markowitz M. et al. [34]	2018	New York	Patient 1	Age 26 y MSM	Oral TDF/FTC Daily	5 months	Time between test negative and positive: 5 months 4th-generation test positive, 3rd-generation test negative; HIV-RNA < 20 cp/mL detected	K65R, M184V K103S, E138Q, Y188L performed on proviral DNA	Yes: excellent adherence; TDF-DP level in hair 0.0448 ng/mg and TDF level in DBS 1478 fmol/punch; results were consistent with high (daily) level of adherence over the preceding 6 to 8 weeks
Hughes J.M. et al. [35]	2021	Canada	Patient 1	Age 60 y MSM	Oral TDF/FTC Daily	16 months	Time between test negative and positive: 2 months 4th-generation test positive and WB positive; HIV-RNA negative; after 2 days, VL 90 cp/mL; after 3 days, VL 227 cp/mL; after 1 month, VL 6949 cp/mL	Wild-type	Yes: reported excellent adherence to PrEP, which was confirmed by pharmacy dispensing records A DBS collected on day 3 revealed an intraerythrocytic TDF concentration consistent with daily dosing
Spinelli M.A. et al. [36]	2021	San Francisco	Patient 1	Age 44 y MSM	Oral TDF/FTC Daily	20 months	Time between test negative and positive: 2 months AHI symptoms (headache, sore throat, and chills) 4th-generation test positive and WB positive; 3rd-generation test negative; HIV-RNA positive	K70N, M184V, V179E, and P225H	Yes: high self-reported PrEP adherence The TDF concentration in the DBS sample collected on the day of HIV treatment initiation was consistent with estimated daily adherence to TDF/FTC over the preceding 6 weeks The TDF level in the proximal 1 cm of hair, corresponding to the 4-week period prior to antiretroviral therapy initiation, was consistent with dosing 7 days a week The TDF hair concentration, corresponding to 4–8 weeks prior to sample collection, was consistent with adherence 5–6 times weekly
Cohen S.E. et al. [19]	2019	San Francisco	Patient 1	Age 21 y	Oral TDF/FTC Daily	13 months	Time between test negative and positive: 3 months 3rd-generation test negative; HIV-RNA positive	RT: 184V, 74V, 100I, 103N	Yes: good self-reported adherence. Segmental hair analysis of TDF concentrations measured in 1 cm segments of hair from the scalp indicated high adherence to PrEP in each of the 6 months before HIV diagnosis Concentrations of TDF (1012 fmol/punch) and emtricitabine triphosphate (0.266 fmol/punch) in a DBS indicated high adherence over the preceding 6 weeks

List of abbreviations. PrEP: pre-exposure prophylaxis; AHI: acute HIV infection; y: years; WB: Western blot; HBV: hepatitis B virus; MSM: men having sex with men; IDU: injection drug user; TGW: transgender women; TGM: transgender men; PWID: people who inject drugs; FSW: female sex workers; TDF: tenofovir disoproxil fumarate; TDF/FTC: tenofovir disoproxil fumarate/emtricitabine; INSTI: integrase strand transfer inhibitor; DBS: dried blood spot; RT: reverse transcriptase; NA: not available.

**Table 2:** Observational studies reporting breakthrough HIV infection in PrEP users.

Study		Location	Population	PrEP Regimen	Subjects on PrEP	Seroconversions	Breakthrough Infections	Adherence	Symptoms	Median Time between Positive and Negative Test Results
Molina J.M. et al. [37]	Cohort study, 2017	France	MSM	Oral TDF/FTC On-demand	361	1/361	0/361	By pill count and plasma drug detection	*n* = 1 (influenza-like syndrome)	NA
Mboup A. et al. [38]	Cohort study, 2018	Africa (Benin)	FSW	Oral TDF/FTC Daily	258	2/258	0/258	Self-reported and by plasma drug detection	NA	NA
Noret M. et al. [39]	Cohort study, 2018	France	MSM	Oral TDF/FTC Daily and on-demand	1049	3/1049	0/1049	Self-reported and by plasma drug detection	1/3	NA
Siguier M. et al. [40]	Cohort study, 2019	France	MSM	Oral TDF/FTC Daily and on-demand	2774	4/2774	2/2774	Self-reported	NA	Patient 1: 1 month Patient 2: 2 months
Tassi M.F. et al. [41]	Cohort study, 2021	France	MSM	Oral TAF/FTC and TDF/FTC Daily and on-demand	9893	29/9893	18/9893	Self-reported	NA	180 days (IQR 124–490)
Molina J.M. at al. [42]	Cohort study, 2022	France	MSM	Oral TDF/FTC Daily and on-demand	3056	6/3056	0/3056	Self-reported	NA	NA
Jourdain H. [43]	Case-control study, 2022	France	MSM	Oral TDF/FTC Daily and on-demand	28,352	266/28,352	260/28,352	Not evaluated	NA	NA
Dibatè S. et al. [44]	Cohort study, 2023	West Africa	MSM	Oral TDF/FTC Daily and on-demand	204	4/204	3/204	Self-reported	NA	NA

List of abbreviations. MSM: men having sex with men; FSW: female sex worker; TDF/FTC: tenofovir disoproxil fumarate/emtricitabine; NA: not available.

**Table 3:** Randomized clinical trials reporting breakthrough HIV infection in PrEP users.

Study	Year	Location	Population	PrEP Regimen	Subjects on PrEP	Seroconversions	Breakthrough Infections	Adherence	Symptoms	Median Time between Positive and Negative Test Results
Grant R.M. et al. (iPrEx) [5]	2011	Peru, Ecuador, South Africa, Brazil, Thailand, and the United States	MSM (99%) and TGW (1%) Age > 18 years	Oral TDF/FTC vs. placebo Daily	1251	36/1251	3/1251	By pill count and plasma drug detection	5 (upper respiratory tract infection)	35 days (IQR 28–56)
Baeten J.M. et al. (Partners PrEP Study) [45]	2012	Kenya and Uganda	Heterosexual serodiscordant couples (negative partner: 62% male) Age > 18	Oral TDF/FTC vs. TDF vs. placebo Daily	3163	30/3163	9/3163	By pill count and plasma drug detection	NA	NA
Van Damme L. et al. (FEM-PrEP) [46]	2012	South Africa	Women Age: 18–35 years	Oral TDF/FTC vs. placebo Daily	1062	33/1062	4/1062	Self-reported and by pill count and plasma drug detection	NA	NA
Choopanya K. et al. (Bangkok Tenofovir Study) [47]	2013	Thailand	PWID Age 20–60	Oral TDF vs. placebo Daily	1204	17/1204	3/1204	By pill count and plasma drug detection	NA	NA
Wei X. et al. (CARPISA 004 Study) [48,49]	2014	South Africa	Women Age 18–40 years	Topical gel Randomized to 1% tenofovir hydroxyethycellulose gel arm or placebo Daily	445	28/445	7/445	By vaginal drug detection >2 ng/mL	NA	33 (IQR 14–77)
Molina et al. (IPERGAY) [50]	2015	Canada and France	MSM and TGW Age > 18 years	Oral TDF/FTC on-demand vs. placebo	206	2/206	0/206	By pill count and plasma drug detection	NA	NA
Baeten J.M. et al. (Partners PrEP Continuation Study) [51]	2015	Kenya and Uganda	Heterosexual serodiscordant couples (negative partner: 62% male) Age > 18	Oral TDF/FTC vs. TDF vs. placebo Daily	4410	52/4410	14/4410	By plasma drug detection	NA	NA
McCormack S. et al. (PROUD) [10]	2016	England	MSM Age > 18 years	Oral TDF/FTC Daily	544	23/544	0/544	Self-reported	NA	NA
Bekker L.G. et al. (HPTN 067/ADAPT Cape Town Trial) [52,53]	2017	Cape Town (South Africa)	Women or TGM Age > 18	Oral TDF/FTC Daily or on-demand	178	6/178	0/178	By electronic dose monitoring with app and plasma drug detection	NA	NA
Hosek S.G. et al. (Project PrEPare) [54]	2017	USA	MSM Age 15–17 years	Oral TDF/FTC Daily	78	3/78	0/78	By adherence follow-up questionnaire and plasma drug detection	NA	NA
Grant R.M. et al. (067/ADAPT Study) [52,55]	2018	Thailand (Bangkok) New York (Harlem)	MSM and TGW	Oral TDF/FTC Daily vs. on-demand	178	4/178	0/178	By electronic dose monitoring with app and plasma drug detection	NA	NA
Grinsztejn B. et al. [56] (PrEP Brazil)	2018	Brazil	MSM and TGW Age > 18 years	Oral TDF/FTC Daily	375	2/375	0/375	By plasma drug detection	NA	NA
Mansoor L.E. et al. [57] (CARPISA 008 Study)	2019	South Africa	Women Age 18–40 years	Topical gel Randomized to 1% tenofovir hydroxyethycellulose gel arm or placebo Daily	189	12/189	2/189	By drug detection in genital fluid	NA	NA
Koss C.A. et al. [58] (The SEARCH study)	2020	Kenya and Uganda	Heterosexual Age > 15 years	Oral TDF/FTC TDF/3TC (due to limitations in TDF/FTC supply) Daily vs. no PrEP	3489	25/3849	7/3849	Self-reported and detection of tenofovir concentrations in hair	NA	NA
Irungu E.M. et al. [59]	2021	Kenya	Serodiscordant couple Age > 18	Oral TDF/FTC, TDF, or TDF/3TC Daily	4898	6/4898	0/4898	Adherence: by plasma drug detection	NA	NA
Grulich A.E. et al. [60] (EPIC—NSW)	2021	New South Wales	MSM and TGW Age > 18	Oral TDF/FTC Daily	9596	30/9596	0/9596	By pill counting	NA	NA
Mujugira A. et al. [61]	2022	Uganda	FSW	Oral TDF Randomized to HIV self-testing or in clinic	110	1/110	0/110	By pill counting	NA	NA (at 9-month visit)
Landovitz R.J. et al. [62] (HPTN 083)	2022	Argentina, Brazil, Peru, the USA, South Africa, Thailand, and Vietnam	MSM and TGW Age > 18 years	Long-acting (LA) CAB LA: once every 8 weeks CAB oral (lead-in) Daily TDF/FTC daily Randomized to CAB (oral tablet lead-in phase—LA) vs. TDF/FTC	4566	58/4566 TDF/FTC *n* = 42 CAB *n* = 16	6/4566 CAB *n* = 4 TDF/FTC *n* = 2	By plasma drug detection	NA	NA
Nair G. et al. [63] (MTN-034/REACH)	2023	South Africa, Uganda, and Zimbabwe	Young women	Oral/topical (vaginal ring) Dapivirine ring or TDF/FTC Daily	247	4/247 dapivirine ring 2/4 oral PrEP 2/4	0/247	Self-reported, questionnaire, and by plasma drug detection	NA	NA
Kinuthia J. et al. [64]	2023	Kenya	Pregnant women: PrEP during post-partum period in those at high risk of HIV acquisition Age > 15 years	Oral TDF/FTC Daily	2197	16/2197	NA	NA	NA	NA
Dumchev et al. [65]	2023	Ukraine	PWID	Oral TDF/FTC Daily	199	1/199	0/199	Self-reported and by plasma drug detection	NA	3 months

List of abbreviations. PrEP: pre-exposure prophylaxis; MSM: men having sex with men; TGW: transgender women; TGM: transgender men; PWID: people who inject drugs; FSW: female sex worker; TDF: tenofovir disoproxil fumarate; TDF/FTC: tenofovir disoproxil fumarate/emtricitabine; NA: not available.

## Data Availability

Not applicable.
